# Prevalence and predictors of virological failure and quality of life of people with HIV/AIDS at a Municipal Hospital, Volta Region –Ghana: A cross-sectional study

**DOI:** 10.1371/journal.pone.0329346

**Published:** 2026-02-13

**Authors:** Kwaku Gyimah Peprah, Faith Agbozo, Mavis Pearl Kwabla, Worlanyo Tashie, Joyce Berkumwin Der

**Affiliations:** 1 Department of Epidemiology and Biostatistics, Fred N. Binka School of Public Health, University of Heath and Allied Sciences, Hohoe, Ghana; 2 Department of Family and Community Health, Fred N. Binka School of Public Health, University of Heath and Allied Sciences, Hohoe, Ghana; 3 West African Centre for Cell Biology of Infectious Pathogens, Department of Biochemistry, Cell and Molecular Biology, College of Basic and Applied Sciences, University of Ghana, Accra, Ghana; Institute of Tropical Medicine / University of Antwerp, BELGIUM

## Abstract

**Background:**

Despite several interventions to eradicate HIV/AIDS globally, virological failure continues to threaten the goals of antiretroviral therapies (ART) and quality of life (QoL) of people with HIV/AIDS (PWHA). This study aimed to assess the prevalence and predictors of virological failure and determine the QoL of PWHA.

**Methods:**

A cross-sectional study was conducted at the ART clinic of a Municipal Hospital, from June to August 2023, to assess the socio-demographic, medical data, and QoL of PWHA receiving therapy at the clinic. Participants were randomly selected and interviewed: their weight and height were taken and their clinic folders examined to assess virological failure status. Both self-developed structured questionnaire and the WHOQOL-HIV BREF scale (Cronbach alpha = 0.84) were used to assess participants’ data. Bivariate and multiple logistic regression analysis were conducted to determine predictors of virological failure. Also, multiple linear regression was conducted to determine factors influencing QoL of study participants.

**Results:**

A total of 398 participants comprising of 328 (82.41%) females, and with a mean age of 48.2 years (SD ± 11.71 years), were recruited into the study. The prevalence of virological failure was 6.03%. Factors such as forgetting to take ART (Adjusted Odds Ratio (AOR) = 2.87, 95% Confidence Interval (CI) = 1.02, 7.51; *p* = 0.04), being classified as baseline WHO clinical staging II (AOR = 6.20, 95% CI = 1.91, 20.04; *p* = 0.002), and HIV stigmatization (AOR = 3.97, 95% C.I. = 1.1, 14.25; *p* = 0.035) were associated with virological failure. The overall QoL was good (75.35%). Having no comorbidities (Coefficient of Determination (β) = −2.7, *p* < 0.0001), having social support (β = 3.94, *p* < 0.0001) and receiving an average monthly income (β = 2.03, *p* = 0.002) contributed to good QoL.

**Conclusion:**

Virological failure in the municipality exceeded the 5.0% target set by the Joint United Nations Programme, despite majority of the study participants presenting with good QoL. The National AIDS Control Programme should consider long-acting injectable therapy for PWHA struggling to adhere to medication.

## Introduction

The Human Immunodeficiency Virus/Acquired Immune Deficiency Syndrome (HIV/AIDS) menace still remains a public health problem [1,2]. Approximately 37.7 million individuals are living with HIV/AIDS globally, with about 25.7 million cases occurring in sub-Saharan Africa [3]. In Ghana, about 334,095 people were estimated to be living with HIV/AIDS in the year 2023 [4]. Several interventions have been implemented in healthcare systems to reduce the burden of HIV/AIDS and subsequently control the HIV epidemic [1,5–7]. Despite these interventions, virological failure continues to impede the health and quality of life (QoL) of people with HIV/AIDS (PWHA) [8].

Antiretroviral therapy (ART) is primarily used to achieve long-term suppression of HIV replication and prolong the life of people with HIV/AIDS (PWHA) [2,9]. ART improves quality of life (QoL) and reduces HIV-associated morbidity and mortality among PWHA [10,11]. The Joint United Nations Programme on HIV/AIDS (UNAIDS), as part of its mandate to end HIV globally, is determined to ensure that 95% of the total population know their HIV status, 95% of people with HIV/AIDS initiates ART, and ensure that 95% of PWHA on therapy achieve viral suppression by the end of 2030 [12].

In Ghana, about 1.66% of the adult population are living with HIV/AIDS whilst about 1.50% of adult population in the Volta Region are living with HIV/AIDS [13]. Although the regional HIV prevalence is slightly lower than the national prevalence, the burden of HIV in the Volta Region remains a public health concern. The advent of new generation ART has been instrumental in reducing HIV replication among PWHA. However, virological failure continues to threaten the goals of ART [14]. Virological failure is a type of HIV treatment failure where the viral load of a person with HIV/AIDS fails to fall below 1000 copies/mL despite taking ART for at least 6 months [15,16]. This treatment failure contributes to high HIV mortality, drug resistance and poor QoL among PWHA [17].

QoL is the feeling of overall life satisfaction, as determined by the mentally alert individual whose life is being evaluated, and it reviews the physical health, social support, financial security, spiritual and emotional well-being of these individuals [18]. QoL is an excellent prognostic indicator for diseases and its application can be used to support decision making which are needed to improve the health of PWHA [19]. Virological failure worsens the health of PWHA by increasing their viral load and reducing the body’s immunity [20]. The reduction in immunity facilitates the development of opportunistic infection, drug resistance and other psychiatric disorders such as depression and anxiety [21]. These worsen the QoL of PWHA [22]. Although the prevalence of virological failure has been determined across several countries in Africa, only few studies have achieved 95% viral suppression rate as established by UNAIDS [17,23,24]. In Ghana, several studies suggest that virological failure continues to impede the goals of ART and UNAIDS [12,25–27].

Ketu South Municipality is among the municipalities in the Volta Region with a high HIV/AIDS burden, noted for risky sexual behaviors such as transactional sex [13]. Despite this assertion, there appears to be paucity of data on virological failure and QoL of PWHA in the municipality. Few researchers have conducted similar works in other municipalities, however, the social, cultural and behavior differences existing among communities and the need to strengthen HIV interventions in the municipality warrant the need to conduct this study [25,28,29]. Therefore, this study aimed to determine the prevalence and predictors of virological failure and QoL of PWHA in the Ketu South Municipality.

## Methods

### Study setting

A hospital-based cross-sectional study was conducted in the Ketu South Municipality of the Volta Region of Ghana from June to August 2023. The municipality has a total land area of about 261 square kilometers, with a population of 253,122 as reported by the 2021 national population and housing census, and shares border with the Republic of Togo [30]. Despite the municipality’s population being composed of farmers and traders, there is also the presence of high-risk behaviors, such as transactional sex, which is commonly observed among border towns [31,32]. Participants were recruited from the ART clinic of the municipal hospital, which serves as the major referral hospital for neighboring health facilities.

### Eligibility criteria

PWHA aged 18 years and above who had been on ART for at least 6 months and had at least one documented viral load measurement were considered for recruitment into the study. Participants who did not have complete medical record prior to the study were excluded. Similarly, individuals who did not give written informed consent were excluded from the study.

### Study design and sampling technique

The study employed a cross-sectional design among PWHA receiving care at the ART clinic. Eligible participants with complete medical records were considered for enrolment into the study. This design enabled the collection of secondary data from medical records as well as primary data through face-to-face interviews. A simple random sampling technique was used to select participants from the HIV population attending the ART clinic of the municipal hospital. This sampling method reduces selection bias and ensures that every eligible client has an equal opportunity of being recruited into the study. Consequently, the findings from this study can be generalized to the wider HIV population who attend the clinic. Specifically, the lottery method of simple random sampling was employed. During the sampling process, unique identification numbers were assigned to all PWHA who visited the ART clinic on clinic days. Each identification number was written on a piece of paper, folded, and placed in a bowl. The papers were thoroughly mixed for about one minute, after which numbers were randomly drawn without replacement until the required daily sample size was achieved. Individuals corresponding to the selected numbers were then approached, and those who provided written informed consent were recruited into the study. Data were collected on all eligible participants on the same day they were enrolled in the study. This procedure was repeated on each clinic day throughout the seven-week data collection period. The ART clinic operates twice a week, with an average of 50 clients reviewed per clinic day. The study aimed to recruit 30 participants during each clinic session. To estimate the data collection period, the total sample size of 398 was divided by the expected weekly recruitment capacity of 60 participants. This calculation indicated that approximately seven weeks were needed to complete data collection. With two clinic days per week, the weekly target of 60 participants was split evenly, resulting in a daily recruitment quota of 30 participants.

### Sample size calculation

The Cochran sample size formula was used to calculate the sample size for this study. Using a 95% confidence level, 5% sample error, 1.96 standard error and a prevalence of virological failure at 38%, the sample size was calculated to be 362 [33]. A 10% non-response rate was applied to this value, resulting in a final sample size of 398 participants.

### Data collection procedure

The study used a structured questionnaire to collect data through face-to-face interviews conducted by the principal researcher in accordance with strict ethical guidelines. Two questionnaires were used for data collection: a pre-tested, self-developed structured questionnaire and another adopted from the World Health Organization. Both questionnaires were administered during the interviews to obtain relevant information from participants. The self-developed questionnaire collected data on drug adherence, social support, stigma, behavioral attitude, clinical characteristics, and socio-demographic information. The WHOQOL-HIV BREF scale, adopted from the World Health Organization (WHO), was specifically used to assess participants’ QoL [34]. After the interviews, each participant’s hospital folder and electronic medical database were reviewed to obtain viral load results and other relevant medical information. The height (in metres) and weight (in kilograms) of participants were also measured to determine their body mass index (BMI). These data were collected electronically using the Kobo Collect software and exported into Microsoft Excel 2016 for analysis.

### Quality of life instrument

The WHOQOL-HIV BREF scale was used to assess the quality of life (QoL) of study participants. The instrument consists of 31 questions categorized into six domains: physical health, psychological health, level of independence, social relationships, environment and spirituality. Each question is rated on a five-point Likert-type scale reflecting participants’ perceptions. Participants’ responses were scored from 1 to 5, with higher scores indicating better perceived quality of life. Scores were assigned in ascending order from option 1(lowest score) to option 5 (highest score). For selected negatively worded items, reverse scoring was applied so that higher values consistently indicated better QoL. Domain scores were computed by summing the item scores within each domain. Each domain was then divided by the maximum attainable score for that domain and expressed as a percentage. Based on the percentage scores, QoL was categorized as excellent (80–100), good (60–70) and poor (<60) [35]. The internal consistency of the WHOQOL-HIV BREF was assessed using Cronbach’s alpha coefficient (α). The overall QoL scale showed good internal consistency (α = 0.84). However, the physical (α = 0.44), psychological (α = 0.63), social relationships (α = 0.36), environment (α = 0.59), and spirituality (α = 0.42) domains showed low to moderate internal consistency.

### Statistical analysis

The dependent variables were virological failure and QoL, whereas the independent variables were the characteristics of socio-demographics, social support, baseline HIV information, stigma, adherence and behavioral attitude of people with HIV/AIDS. Descriptive analysis was perfomed to determine mean and standard deviation for the continuous variables, and frequencies and percentages for the categorical variables. In the inferencial statistics, bivariate logistic regression was performed to determine predictor variables that were associated with virological failure. Predictor variables with *p*-value less than 0.2 were fitted into the multiple logistic regression analysis. An initial *p*-value threshold of less than 0.2 was used in the bivariate logistic regression to screen the numerous predictor variables. This approach ensured that potential predictors and confounders were included for adjustment in the multiple logistic regression analysis. In the multiple logistic regression, variables with *p*-value less than 0.05 were considered as significant predictors of virological failure. Multiple linear regression analysis was also performed to determine factors that were associated with QoL of participants. At all times, a *p-*value < 0.05 was considered as statistically significant. All analysis were performed using STATA (version 13).

### Ethical consideration

Approval for this study was obtained from the Ethics Review Committee of University of Health and Allied Sciences (UHAS-REC A.5 [1] 22–23). Permission was sought from the Municipal Hospital before accessing the facility for the study. Written informed consent was also obtained from each participant after the objective and rationale of the study had been explained to them.

## Results

### Socio-demographic characteristics of people with HIV/AIDS

A total of 398 people with HIV/AIDS were included in the study, of which 328 (82.41%) were females. Also, 120 (30.15%) participants had no formal education whereas 139 (34.92%) and 100 (25.13%) had completed basic and junior high school respectively. The majority of study participants (193, 48.54%) were married whilst 115 (28.89%) and 44 (11.06%) were widowed and single respectively (Table 1).

**Table 1:** Socio-demographic characteristics of people with HIV/AIDS at a Municipal Hospital, Volta Region.

Variables	Frequency (n)	Percent (%)
**Gender**		
Male	70	17.59
Female	328	82.41
**Age (years)**		
Mean age (years)	48.2 ± 11.71	
< 30	22	5.53
30-39	67	16.83
40-49	135	33.92
50-59	109	27.39
≥ 60	65	16.33
**Marital status**		
Single	34	8.54
Married	193	48.54
Separated	12	3.02
Divorce	44	11.06
Widowed	115	28.89
**Monthly income (GHS)**		
< 500	194	48.74
500-1599	198	49.25
≥ 1600	6	2.01

GHS = Ghana Cedis

### Clinical and adherence characteristics of people with HIV/AIDS

There were 88 (22.1%) study participants with comorbidities such as hypertension, tuberculosis and diabetes; 93 (23.37%) had a history of discontinuing their ART medication; and 262 (65.83%) had kept ART scheduled appointment. Also, 379 (95.23%) were currently taking TDF-3TC-DTG antiretroviral combination therapy. Additionally, 108 (27.14%) participants had ever forgotten to take their ART medication. It was noted that 280 (70.35%) participants had their HIV status disclosed. Only 29 (7.34%) reported to have been stigmatized. This is summarized in Table 2.

**Table 2:** Clinical and adherence characteristics of people with HIV/AIDS at a Municipal Hospital, Volta Region.

Variables	Frequency (n)	Percent (%)
**Baseline WHO Clinical Staging**		
Stage 1	330	82.91
Stage 2	38	9.55
Stage 3	28	7.04
Stage 4	2	0.5
**Final WHO Clinical Staging**		
Stage1	388	97.49
Stage 2	8	2.01
Stage 3	2	0.5
**Current ART Regimen**		
AZT 3TC DTG	19	4.77
TDF 3TC DTG	379	95.23
**Presence of comorbid**		
Yes	88	22.11
No	310	77.89
**Keep scheduled appointment**		
Yes	262	65.83
No	136	34.17
**Have you ever forgotten intake of ART**		
Yes	108	27.14
No	290	72.86
**Forgotten ART intake during weekend**		
Yes	51	12.81
No	347	87.19
**Social support**		
Yes	201	50.5
No	197	49.5
**HIV disclosure**		
Yes	280	70.35
No	118	29.65
**HIV stigma**		
Yes	29	7.29
No	369	92.71

AZT = Zidovudine; 3TC = Lamivudine; EFV = Efavirenz; NVP = Nevirapine; TDF = Tenofivir Disoproxil Fumarate; DTG = Dolutegravir; ART = Antiretroviral Therapy; WHO = World Health Organization.

### Factors associated with virological failure among people with HIV/AIDS

Table 3 presents the results of both bivariate and multiple logistic regression analysis examining variables of participants associated with virological failure. The multiple logistic regression model explained 34% of the variability in virological failure (pseudo coefficient of determination (R^2^) = of 0.3403). People with HIV/AIDS who were grouped in baseline WHO stage 2 were 6 times more likely to develop virological failure compared with their counterparts in WHO stages 1 (AOR = 6.2; 95%CI = 1.91, 20.04; p = 0.002). Also, people with HIV/AIDS who forgot to take their ART medication were 2.78 times more likely to develop virological failure than those who always take their medication (AOR = 2.78; 95%CI = 1.02, 7.51; p = 0.04). People with HIV/AIDS who reported being stigmatized were 2 times more likely to develop virological failure than those who reported not being stigmatized (AOR = 2.11; 95%CI = 1.1, 14.25; p = 0.035).

**Table 3:** Factors associated with virological failure among people with HIV/AIDS at a Municipal Hospital, Volta Region.

Variable	VF(%)	OR	*p*-value(95%CI:L,U)	AOR	*p*-value(95%CI:L,U)
**Age**					
< 30	3(12.51)	1			
30-39	8(33.33)	0.86	0.83(0.21, 3.56)	0.81	0.8(0.15, 4.16)
40-49	5(20.83)	0.24	0.06(0.54, 1.10)	0.25	0.118(0.04, 1.41)
50-59	6(25.0)	0.37	0.18(0.08, 1.60)	0.39	0.289(0.07, 2.18)
≥ 60	2(8.33)	0.2	0.09(0.03, 1.29)	0.19	0.128(0.02, 1.6)
**BMI**					
Underweight	3(12.5)	1			
Normal	13(54.17)	0.37	0.14(0.09, 1.42)	0.34	0.183(0.07, 1.65)
Overweight	8(33.33)	0.5	0.34(0.12, 2.06)	0.55	0.49(0.11, 2.93)
**Baseline WHO stage**					
Stage 1	15(62.50)	1			
Stage 2	6(25.0)	3.94	0.08(1.43, 10.85)	6.2	0.002(1.91, 20.04)
Stage 3	3(12.5)	2.52	0.16(0.68, 9.29)	2.12	0.337(0.45, 9.83)
**ART discontinuation**					
No	16(66.67)	1			
Yes	8(33.33)	2.5	0.03(1.07, 5.84)	2.08	0.156(0.75, 5.73)
**Forgetting ART**					
No	11(45.83)	1			
Yes	13(54.17)	3.47	0.004(1.50, 8.0)	2.78	0.04(1.02, 7.51)
**Stigma**					
No	19(79.17)	1			
Yes	5(20.83)	3.8	0.014(1.30, 11.08)	2.11	0.035(1.1, 14.25)

*p* < 0.05 was considered statistically significant for the variables in the table.

OR= Odds Ratio ART = Antiretroviral therapy, BMI = Body Mass Index, WHO = World Health Organization, AOR = Adjusted Odds Ratio, VF = Virological failure, L = Lower, U = Upper

### Components of quality of life in relation to gender

Table 4 summarizes the various QoL domains in relation to gender. There were significant relationship between overall QoL (*p =* 0.0002), physical domain (*p =* 0.005), psychological (*p <* 0.0001) and environment domain (*p* < 0.0001) in relation to gender. Health satisfaction (*p =* 0.9398), level of independence (*p =* 0.118), social relationships (*p =* 0.1945) and spiritual/religion/personal belief were not significantly related to gender. The overall QoL mean score was 75.35% (SD: 6.77). The physical domain recorded the highest score (86.01%) whilst the environmental domain recorded the least score (62.73%).

**Table 4:** Quality of life components by gender among people with HIV/AIDS at a Municipal Hospital, Volta Region.

Domains of life	Mean	SD	Female	Male	p-value
**Physical Health**	86.01	10.58	85.32	89.21	0.005
Q1. Pain and discomfort	95.52	11.83			
Q2, Problems related to HIV	95.97	10.33			
Q13. Energy for everyday life	72.66	21.86			
Q20. Sleep satisfaction	79.84	17.41			
**Psychological**	78.25	11.27	77.26	82.91	<0.0001
Q4. Enjoy life	66.73	18.74			
Q9. Concentrate well	69.49	20.79			
Q14. Accept bodily appearance	84.12	18.86			
Q23. Self-satisfaction	81.55	13.03			
Q12. Negative feelings	89.34	16.58			
**Level of independence**	71.13	8.49	70.82	72.57	0.118
Q3. Dependence on treatment	48.44	24.89			
Q19. Ability to get around	76.68	16.03			
Q21. Activities of daily living	79.74	12.49			
Q22. Work capacity	79.64	11.83			
**Social relationships**	75.94	9.03	75.67	77.21	0.1945
Q16. Social inclusion	76.38	17.64			
Q24. Personal relationships	79.59	12.61			
Q25. Sexual activity	76.68	12.57			
Q26. Social support	71.1	18.13			
**Environment**	62.73	8.36	61.91	66.61	**<**0.0001
Q10. Physical safety and security	61.35	17.81			
Q11. Physical environments	62.16	16			
Q15. Financial resources	43.51	19.5			
Q17.Availability of information	59.04	18.45			
Q18. Recreation and leisure	41.05	20.27			
Q27.Conditions of living place	78.99	9.67			
Q28. Access to health service	81.25	9.05			
Q29. Transport	74.47	16.29			
**Spirituality**	85.79	10.35	85.49	87.21	0.2058
Q5. Feel of meaningful life	66.68	19.5			
Q6. Receive blame as PWHA	91.3	18.06			
Q7. Concerns about the future	90.8	17.17			
Q8. Death and dying	94.37	13.18			
**Overall quality of life**	75.94	6.77	74.77	78.06	0.0002

*p* < 0.05 was considered statistically significant for the variables in the table. Data is presented as mean. SD = standard deviation.

### Factors associated with quality of life of people with HIV/AIDS

Table 5 presents the results of the multiple linear regression analysis examining the association between participants’ characteristics and overall QoL. The model explained 27.2% of the variance in overall QoL (R^2^ = 0.2717; adjusted R^2^ = 0.2245). Variables such as male gender (β = 0.048, *P* = 0.048) and monthly income (β = 2.03, *p* = 0.002) were positively associated with overall QoL. Also, social support (β = 2.94, *p* < 0.0001) and comorbidity (β = −2.7, *p* < 0.0001) were significantly associated with overall QoL.

**Table 5:** Factors associated with quality of life of people with HIV/AIDS at a Municipal Hospital, Volta Region.

Variables	Coefficient(β)	*P*-value	95% CI(Lower, upper)
**Marital status**			
Married	1.71	0.106	−0.363, 3.783
Separated	−2.841	0.155	−6.758, 1.075
Single	0.959	0.533	−2.061, 3.981
Widowed	0.801	0.466	−1.132, 2.960
**Comorbidity**			
Yes	−2.708	**<**0.0001	−4.209, −1.207
**Education**			
None	−0.009	0.990	−1.544, 1.524
Senior High	0.244	0.863	−2.529, 5.587
Tertiary	2.343	0.220	−1.408, 6.095
**Income (GHS)**			
500-1599	2.037	0.002	0.729, 3.345
1600 or more	4.988	0.094	−0.859, 10.836
**Occupation**			
Self-employed	2.978	0.536	−2.334, 0.911
Unemployed	1.270	0.794	−8.291,10.833
**Social support**			
Yes	3.942	<0.0001	2.696, 5.187

*p* < 0.05 was considered statistically significant for the variables in the table

CI= Confidence Interval, GHS=Ghana Cedis, β= Regression coefficient.

## Discussion

The study determined the prevalence and predictors of virological failure and assessed the QoL of people with HIV/AIDS. The prevalence of virological failure observed in this study was 6.03%, which is slightly higher than the global UNAIDS target of 5.0% [36,37]. However, it is substantially lower than estimates reported in earlier Ghanaian studies by Abubakari et al. (47%), Owusu et al. (19%) and Boakye et al. (24%) [12,25,26]. The relatively low prevalence observed in this study may be attributed to the fact that the majority of participants (97.47%) were receiving dolutegravir-based regimens. Unlike this study, earlier Ghanaian studies reporting higher virological failure rates predominantly involved participants on efavirenz-based first-line therapy. Dolutegravir has been shown to achieve sustained viral suppression, has a high tolerance rate and provides a higher resistance barrier against mutations, with studies from both Botswana and Ghana confirming its superior effectiveness compared to efavirenz in reducing viral load [23,26,38,39]. This shift towards dolutegravir-based treatment in the current study population likely contributed to the lower virological failure rate. In addition to the widespread use of dolutegravir-based treatment, the high rate of HIV status disclosure among participants (70.35%) may also have contributed to the low prevalence of virological failure. Individuals who disclose their HIV status are more likely to receive strong social support, including companionship, coping strategies, financial aid and emotional support [40,41]. Such support not only improves quality of life but also promote better adherence to ART. Improved adherence, in turn, enhances viral suppression and reduces the likelihood of treatment failure.

The study further identified HIV-related stigma, forgetfulness in taking ART and WHO clinical staging II at baseline as significant predictors of virological failure. Participants who reported forgetting to take their ART medications were more likely to develop virological failure compared to those who adhered strictly to treatment. Notably, most instances of forgetfulness occurred during the weekends, suggesting that disruptions in routine activities during this period may contribute to missed doses. Although the present study did not investigate the specific reasons for the forgetfulness in taking ART, previous studies have shown that factors such as being busy, travelling, depression, stress and lack of medication reminders may contribute to poor adherence [42,43]. ART must therefore be taken daily to maintain adequate drug concentrations in the bloodstream and ensure sustained virologic control. Missed doses reduce drug levels to sub-therapeutic thresholds, allowing viral replication. Repeated lapses in adherence may eventually lead to the development of drug-resistant viral strains, thereby reducing treatment effectiveness and increasing the risk of virological failure [43,44]. These findings align with observations in other researchers [14,45,46].

The study also observed that PWHA who are classified under WHO clinical stage II at baseline were more likely to develop virological failure. Individuals in this stage often present with mild clinical symptoms including unexplained weight loss of less than 10% of total body weight, recurrent respiratory infections and other dermatological symptoms prior to antiretroviral therapy initiation [47]. Such individuals are often diagnosed late, and the late diagnosis is associated with a higher risk of HIV transmission, treatment failure, opportunistic infections and increased viral load [48]. This finding aligns with a study from Ethiopia by Desta and colleagues, which observed that WHO clinical staging II was associated with a higher likelihood of virological failure [49]. Similarly, Kityo et al. observed comparable findings among Ugandan children, where WHO clinical staging II significantly contributed to virological failure [50].

The study further found that participants who experienced HIV-related stigmatization were more likely to develop virological failure. This finding is consistent with observations by Hargreaves and colleagues, who reported that internalized HIV stigma was associated with an increased risk of virological failure [51]. Stigma remains a critical barrier to effective HIV management, as it undermines key interventions such as ART adherence, and discourages engagement in voluntary counselling and testing. Stigmatizing experiences can lead to HIV non-disclosure, reduced social support, poor treatment outcomes, all of which contribute to the risk of virological failure [52].

The overall QoL observed among participants was good (75.35%), consistent with findings from Ghana (71.29%) and Indonesia (71.6%) [27,53]. However, the environmental domain recorded the lowest QoL score, a trend observed in studies conducted in Ghana and Portugal [54–56]. Monthly income was identified as a key factor influencing the improved QoL observed in this study. This observation aligns with a study by Ebrahimi-Kalan et al. which reported that low monthly income reduces QoL among PWHA in Iran [57]. Similarly, Jiang and colleagues observed that higher monthly income contributes to better QoL among men who have sex with other men in China [58]. Participants with higher income are better able to meet their health, nutritional and financial needs, which positively influence both immunity and mental well-being.

Social support was also found to positively influence the overall QoL of participants, affecting all domains of life assessed by the WHOQOL-HIV BREF scale. Similar findings were made by Abrefa-Gyan et al. and Birore et al. where social support positively improved the QoL of PWHA in Ghana [59,60]. Social support not only improves mental health, but also provides financial assistance, promotes HIV disclosure and improves drug adherence among PWHA [29,61]. Additionally, the absence of comorbidities contributed to the good QoL observed in this study. Participants without comorbid conditions were free from complications such as renal impairment, drug-drug interactions, and other HIV-related issues. Similar findings by Nigusso et al. and Langebeek et al. attributed higher QoL scores to the absence of comorbidities among PWHA [22,62]. Interestingly, whereas other studies have reported poor QoL among HIV-infected individuals with virological failure, this study observed good QoL among these participants.

### Limitation

The use of a cross-sectional study introduced minimal selection bias to the study, however, it enabled efficient data collection within a short time frame, eliminating the need for participant follow-up.

## Conclusion

Virological failure in the municipality was high, exceeding the 5.0% target set by the UNAIDS. Factors such as forgetfulness in taking ART, WHO clinical staging II and HIV stigmatization contributed to the observed virological failure. Therefore, it is recommended that the Government of Ghana in collaboration with the National AIDS Control Programme (NACP), consider implementing long-acting injectable therapy in routine management of HIV/AIDS to improve medication adherence and reduce stigma among PWHA. Despite the high prevalence of virological failure, the majority of study participants presented with good QoL. Factors such as presence of social support, monthly income and the absence of comorbidities contributed to the improved QoL observed. Routine assessment of QoL in ART clinics is crucial for improving health outcomes among PWHA. Strengthening social support systems, including peer support groups within ART clinics and broader community networks, could also provide emotional stability and security for PWHA. Additionally, the NACP should collaborate with financial donors, government agencies and other social welfare organizations to establish employment opportunities and provide financial and material assistance to economically disadvantaged people with HIV/AIDS.

## Supporting information

S1 Fig Prevalence of virological failure among people with HIV/AIDS in a municipal hospital, with 93.97% of participants achieving viral suppression. (DOCX)

S1 File Raw data collected from study participants. (XLSX)

S2 File Study questionnaire administered to participants, including questions on adherence, stigma, quality of life, and socio-demographic information. (DOCX)

## Abbreviations

β: Regression coefficient
3TC: Lamivudine
AIDS: Acquired Immune Deficiency Syndrome
AOR: Adjusted Odds Ratio
ART: AntiRetroviral Therapy
AZT: Zidovudine
BMI: Body Mass Index
CI: Confidence Interval
DTG: Dolutegravir
EFV: Efavirenz
GHS: Ghana Cedis
HIV: Human Immunodeficiency Virus
NACP: National AIDS Control Programme
NVP: Nevirapine
PWHA: People With HIV/AIDS
QoL: Quality of Life
R^2^: Coefficient of Determination
SD: Standard Deviation
SSA: Sub-Saharan Africa
TB: Tuberculosis
TDF: Tenofivir Disoproxil Fumarate
UNAIDS: Joint United Nations Program on HIV/AIDS
WHO: World Health Organization
WHOQOL: World Health Organization Quality of Life
