# Supplementary material for: Prevalence and predictors of virological failure and quality of life of people with HIV/AIDS at a Municipal Hospital, Volta Region –Ghana: A cross-sectional study
Source: PLoS One. 2026 Feb 13;21(2):e0329346. doi: 10.1371/journal.pone.0329346 (PMC12904377; doi:10.1371/journal.pone.0329346)
Supplement: S1 Fig — (DOCX) [file pone.0329346.s001.docx]

## Prevalence of virological failure among study participants

The proportion of HIV- infected adults with virological failure is depicted in figure 1. The proportion of study participants with virological failure was 6.03% whilst 93.97% had their viral load suppressed.

**
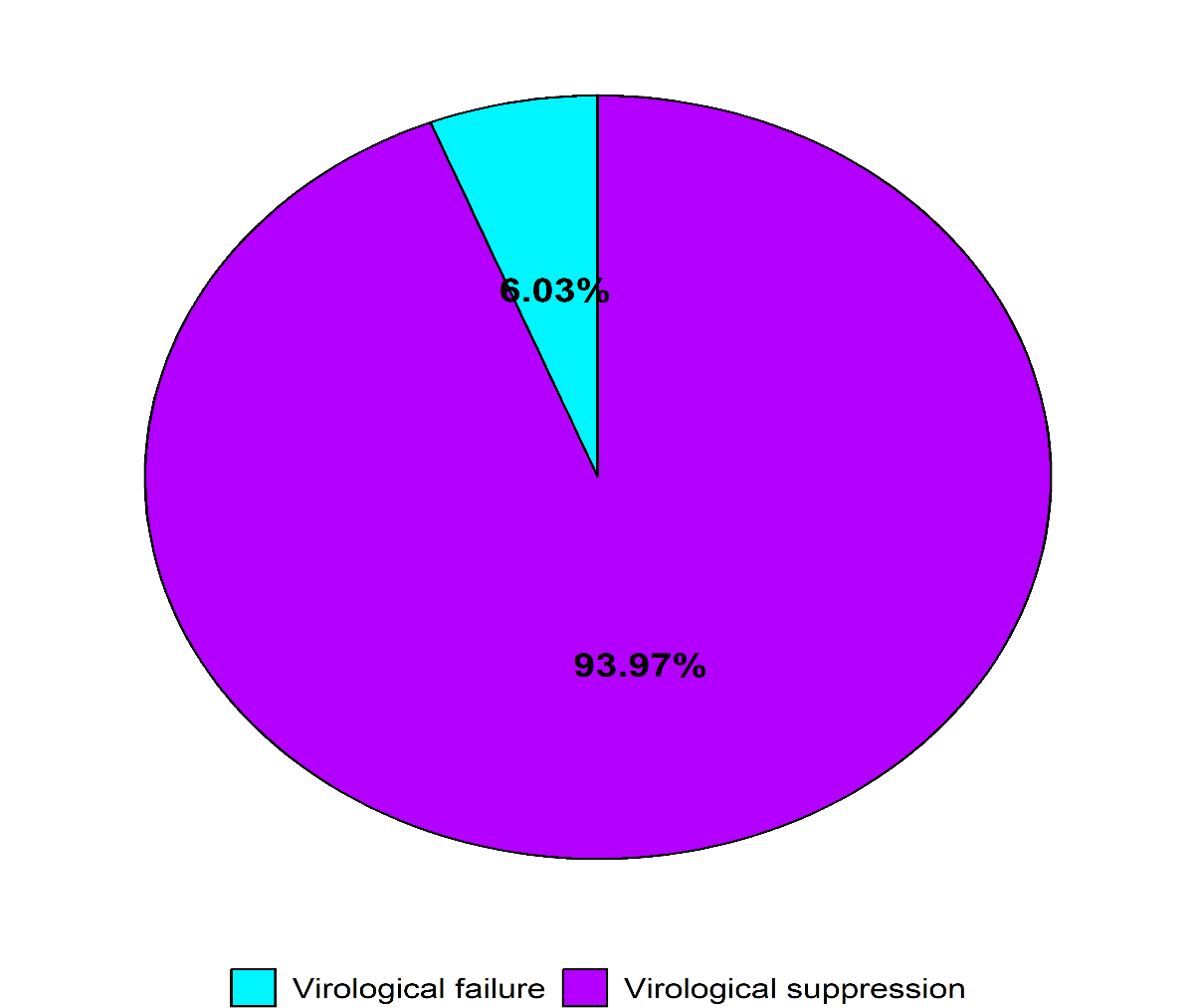
**

Fig 1: Prevalence of virological failure among HIV-infected adults on ART
