# Supplementary material for: Prevalence and predictors of virological failure and quality of life of people with HIV/AIDS at a Municipal Hospital, Volta Region –Ghana: A cross-sectional study
Source: PLoS One. 2026 Feb 13;21(2):e0329346. doi: 10.1371/journal.pone.0329346 (PMC12904377; doi:10.1371/journal.pone.0329346)
Supplement: S2 File — (DOCX) [file pone.0329346.s003.docx]

**Research Questionnaire**

**Topic:** Prevalence and predictors of Virological Failure among HIV-Infected Adults on Anti-Retroviral Therapy in the Ketu South Municipality

1. **Patient demographic information**

Identification number_____________ Date of visit___/_____/____ H/No: ___________________ Health Facility___________________________ Age______ Date of birth ___/____/____

Town of Residence__________________ Sex: (a) Male (b) Female

Marital status: (a) Single (b) Married (c) Divorced (d) Separated (e) Widowed

Religion: (a) Christian (b) Muslim (c) Others________________

Occupation: ____________________

Educational status: (a) None (b) Basic (c) Secondary (d) Secondary (e) Tertiary

Ethnicity: (a) Ewe (b) Akan (c) Ga (d) Others____________________

Monthly income in GHC: _____________________

Weight (Kg): ____________________ Height (cm): _____________________

1. **Baseline data**

1. Date of HIV confirmation: ___/___/____ 2. Age at First start of ART __________

3. Baseline BMI at start of ART (Kg/$m^{2}$) _____________

4. Baseline viral load measurement­­­_________ 5. Current viral load measurement________

6. WHO HIV stage prior to ART enrolment: (a) Stage 1(b) Stage 2 (c) Stage 3 (d) Stage 4

7. Current WHO HIV stage (a) Stage 1 (b) Stage 2 (c) Stage 3 (d) Stage 4

8. Baseline Functional status: (a) Working (b) Ambulatory (c) Bedridden

9. Current Functional status: (a) Working (b) Ambulatory (c) Bedridden

10. How many years have you been on ART? ______________________

11. Baseline first line ART regimen_______________

12. Current ART regimen___________________

13. Has ART regimen ever been changed to an alternative first line regimen? (a) No (b) Yes

14. If answer to Q13 is yes, how many times has ART regimen changed to alternative first-line ART? _____________

15. If yes to question 13, why was it changed?

(a) Drug side effect (b) Non-availability (c) Virological failure (d) Others__________________

16. Has first line regimen ever been changed to second line ART regimen? (a) No (b) Yes

17. If answer to Q16 is yes, which second line regimen was administered? __________________

18. If yes to question 16, why was it changed?

(a) Drug side effect (b) Non-availability (c) Virological failure (d) Others__________________

19. Are you given Cotrimoxazole prophylaxis? (a) No (b) Yes

20. Are you given Fluconazole prophylaxis? (a) No (b) Yes

21. Are you given INH (isoniazid) prophylaxis? (a) No (b) Yes

22. Do you have any of these Comorbid illness? Please tick in the boxes [ ] Diabetes mellitus [ ] Hypertension [ ] Pneumonia [ ] Hepatitis B [ ] None [ ] Others_____________________

23. Do you currently have any Opportunistic infections? (a) No (b) Yes

24. If answer to Q21 is yes, what are they ________________________

25. Have you ever developed Tuberculosis (TB) since the start of ART? (a) No (b) Yes

26. Do you have TB? (a) No (b) Yes

27. If your answer to Q23 is yes, do you use TB medications such as Rifampicin, Pyrazinamide, isoniazid, Ethambutol hydrochloride? (a) No (b) Yes

28. Do you have a history of ART discontinuation? (a) No (b) Yes

29. If answer to Q25 is yes, indicate length of time of discontinuation _____________

30. Do you keep scheduled ART clinical appointments? (a) No (b) Yes

31. How often do you miss ART clinical appointment? Please tick in the box [ ] Never [ ] Rarely [ ] Sometimes [ ] Often [ ] Always

32. Do you spend longer time at the ART clinic? (a) No (b) Yes

33. How many hours do you spend at the ART clinic? _________________

**C. Adherence to ART medication**

1. Do you always take your medication at the appropriate time? (a) No (b) Yes

2. When you feel bad, do you ever discontinue taking your medication? (a) No (b) Yes

3. Have you ever forgotten to take your medication? (a) No (b) Yes

4. Have you ever forgotten to take your medication during the weekend? (a) No (b) Yes

5. In the last week, how many times did you fail to take your prescribed dose? (a) Never (b) 1-2 times (c) 3-5 times (d) 6-10 times (e) more than 10 times

6. Do you fail to take ART medication because of side effect of drug? (a) No (b) Yes

7. Do you fail to take ART medication because of forgetfulness? (a) No (b) Yes

8. Do you fail to take ART medication because of disappointment? (a) No (b) Yes

9. Do you fail to take ART medication because of social stigma? (a) No (b) Yes

11. Do you fail to take ART medication because of depression? (a) No (b) Yes

12. Do you fail to take ART medication because you feel better? (a) No (b) Yes

13. Do you fail to take ART medication because of lack of family care? (a) No (b) Yes

14. Do you fail to take ART medication because of alcohol use? (a) No (b) Yes

15. Are there any other reasons why you fail to take ART medications? ______________________________________________________________

16. Do you take traditional medicine in addition to ART? (a) No (b) Yes

**D. Social Support**

1. Do you have any support in taking medication and managing the disease? (a) No (b) Yes

2. If yes to question 1, from whom do you get social support in managing with the disease? (a) Family members (b) Friends (c) Peer educators (d) Social organizations

3. If yes to question 1, how do they support you? (a) Assist in taking medication (b) Emotional support (c) Financial support (d) Information support (e) Others________________

4. If yes to question 1, are you satisfied with the social support given you?

(a) Highly satisfied (b) Averagely satisfied (c) Not satisfied (d) Indifferent

**E. Stigma**

1. Have you disclosed your status to anyone? (a) No (b) Yes

2. If yes to question 1, who did you disclose your status to? (a) Family member (b) Friends (c) Peer educator (d) Others______________________

3. If yes to question 1, have you been able to relate well with them after disclosure? (a)No (b)Yes

4. If yes to question 1, has disclosure affected your adherence to medication? (a) No (b) Yes

5. Are you stigmatized by family and friends? (a) No (b) Yes

**F. Behavioral Attitude**

1. Were you having a partner prior to HIV infection? (a) No (b) Yes

2. If answer to Q1 is yes, have you disclosed your status to your partner? (a) No (b) Yes

3. If answer to Q2 is yes, what is the HIV status of your partner? (a) Negative (b) Positive (c) No idea

4. If answer to Q3 is positive, what is the couple’s date of ART start? ________________

5. Do you have any history of substance abuse? Please tick in the box

[ ] None [ ] Alcohol [ ] Smoking [ ] Prescription abuse [ ] Others___________________

6. Do you have multiple sex partners? (a) No (b) Yes

7. How many times do you have sex in a month? ____________

8. Do you use condom at all times during sex? (a) No (b) Yes

|  | | Not at all | A little | A moderate amount | Very much | An extreme amount |
| --- | --- | --- | --- | --- | --- | --- |
| 1 | To what extent do you feel that physical pain prevents you from doing what you need to do? | 1 | 2 | 3 | 4 | 5 |
| 2 | How much are you bothered by any physical problems related to your HIV infection? | 1 | 2 | 3 | 4 | 5 |
| 3 | How much do you need any medical treatment to function in your daily life? | 1 | 2 | 3 | 4 | 5 |
| 4 | How much do you enjoy life? | 1 | 2 | 3 | 4 | 5 |
| 5 | To what extend do you feel your life to be meaningful? | 1 | 2 | 3 | 4 | 5 |
| 6 | To what extent are you bothered by people blaming you for your HIV status? | 1 | 2 | 3 | 4 | 5 |
| 7 | How much do you fear the future? | 1 | 2 | 3 | 4 | 5 |
| 8 | How much do you worry about death? | 1 | 2 | 3 | 4 | 5 |
| 9 | How well are you able to concentrate? | 1 | 2 | 3 | 4 | 5 |
| 10 | How safe do you feel in your daily life? | 1 | 2 | 3 | 4 | 5 |
| 11 | How healthy is your physical environment? | 1 | 2 | 3 | 4 | 5 |

**Questionnaire for accessing Quality of Life among HIV adult patient**

1. The following questions ask about how much you have experienced certain things in the last two weeks.

The following question refers to how often you have felt or experienced certain things in the last two weeks.

|  | | Never | Seldom | Quite often | Very often | Always |
| --- | --- | --- | --- | --- | --- | --- |
| 12 | How often do you have negative feelings such as blue mood, despair, anxiety and depression? | 1 | 2 | 3 | 4 | 5 |

1. The following questions ask about how completely you experience or were able to do certain things in the last two weeks.

|  | | Not at all | A little | Moderately | Mostly | Completely |
| --- | --- | --- | --- | --- | --- | --- |
| 13 | Do you have enough energy for everyday life | 1 | 2 | 3 | 4 | 5 |
| 14 | Are you able to accept your bodily appearance | 1 | 2 | 3 | 4 | 5 |
| 15 | Have you enough money to meet your needs | 1 | 2 | 3 | 4 | 5 |
| 16 | To what extent do you feel accepted by the people you know | 1 | 2 | 3 | 4 | 5 |
| 17 | How available to you is the information that you need in your daily life | 1 | 2 | 3 | 4 | 5 |
| 18 | To what extent do you have the opportunity for leisure activities | 1 | 2 | 3 | 4 | 5 |
| 19 | How well are you able to get around | 1 | 2 | 3 | 4 | 5 |

1. The following questions ask you how good or satisfied you have felt about various aspects of your life over the last two weeks

|  | | Very dissatisfied | Dissatisfied | Neither satisfied or dissatisfied | Satisfied | Very satisfied |
| --- | --- | --- | --- | --- | --- | --- |
| 20 | How satisfied are you with your sleep | 1 | 2 | 3 | 4 | 5 |
| 21 | How satisfied are you with your ability to perform your daily living activities | 1 | 2 | 3 | 4 | 5 |
| 22 | How satisfied are you with your capacity for F | 1 | 2 | 3 | 4 | 5 |
| 23 | How satisfied are you with yourself | 1 | 2 | 3 | 4 | 5 |
| 24 | How satisfied are you with your personal relationship | 1 | 2 | 3 | 4 | 5 |
| 25 | How satisfied are you with your sex life | 1 | 2 | 3 | 4 | 5 |
| 26 | How satisfied are you with the support you get from your friends | 1 | 2 | 3 | 4 | 5 |
| 27 | How satisfied are you with the conditions of your living place | 1 | 2 | 3 | 4 | 5 |
| 28 | How satisfied are you with your access to health service | 1 | 2 | 3 | 4 | 5 |
| 29 | How satisfied are you with your transport | 1 | 2 | 3 | 4 | 5 |
| 30 | How satisfied are you with your health | 1 | 2 | 3 | 4 | 5 |

|  | Much better | Somewhat better | About the same | Somewhat worse | Much worse |
| --- | --- | --- | --- | --- | --- |
| 31. Compared to one year ago, how would you rate your health in general now? | 1 | 2 | 3 | 4 | 5 |
